# Airway obstruction during pneumonectomy using a single lumen tube: A case report

**DOI:** 10.1097/MD.0000000000019736

**Published:** 2020-04-17

**Authors:** Shill Lee Son, Woo-Shik Kim, Miyoung Kwon, Yeon-Jin Moon

**Affiliations:** a Department of Anesthesiology and Pain Medicine’; b Department of Thoracic and Cardiovascular Surgery, National Medical Center, Seoul, Korea.

**Keywords:** airway obstruction, endotracheal intubation, general anesthesia, pneumonectomy

## Abstract

**Rationale:**

Endotracheal intubation is an essential step for airway management during general anesthesia. When surgeons carry out thoracic surgery such as pneumonectomy, they usually request lung isolation to secure a clear surgical view. A double lumen endotracheal tube is used for lung isolation in routine thoracic surgeries.

**Patient Concerns:**

A 56-year-old man was previously diagnosed with left Aspergilloma, a tuberculosis destroyed lung, and diabetes mellitus. According to his chest x-ray and chest computed tomography, his left lung was nearly collapsed, and the result of a pulmonary function test was severely restricted. The patient's diffusing capacity for carbon monoxide was 63% and predicted postoperative forced expiratory volume in 1 second was 23.5%

**Diagnoses:**

Due to his previous history, radiologic findings and laboratory test results, he was diagnosed with left Aspergilloma and tuberculosis destroyed lung.

**Interventions:**

Due to recurrence of Aspergilloma in his left lung, the patient was scheduled for a left pneumonectomy. Since the patient's partial oxygen concentration was adequate despite his left lung being nearly totally collapsed, we thought that we would be capable of performing the pneumonectomy using a single lumen tube (SLT). For a better surgical view, we planned lung isolation via insertion of a SLT deep into the bronchus.

**Outcomes:**

During pneumonectomy, after tracheal suction was performed, we tried a lung recruitment maneuver. Suddenly end-tidal carbon dioxide did not show on the monitor. The patient's blood pressure dropped and heart rate was decreasing. We thought that cardiopulmonary resuscitation was needed and an approximately 2 cm sized hematoma was removed from the endotracheal tube after vigorous suctioning. After getting rid of the hematoma, we changed the single tube to a double lumen tube (DLT).

**Lessons:**

This case led us to the conclusion that a DLT should be used for safety when carrying out thoracic surgery. We report a rare case of an airway obstruction using a SLT during pneumonectomy.

## 1 Introduction

Anesthetic management should be planned to protect the airway and ensure correct ventilation as well as patient safety.^[1]^ Endotracheal intubation is an essential step for airway management during general anesthesia. Therefore, obstruction of an endotracheal tube can be a potentially life-threatening event.^[2]^ If surgeons have to perform thoracic surgery, especially pneumonectomy, they usually request lung isolation to secure a clear surgical view and prevent soiling of the dependent lung. Lung isolation is especially important for patient safety as the mortality rate following pneumonectomy exceeds that of lobectomy. This is because of postoperative cardiac complications and acute lung injury.^[3]^ Lung isolation techniques are commonly used to facilitate surgical exposure and provide one-lung ventilation in patients undergoing a variety of intrathoracic surgical procedures.^[4]^ Lung isolation is currently achieved by two primary methods: a double-lumen endotracheal tube or a bronchial blocker. Double lumen tube (DLT) can facilitate the procedure by collapsing the nondependent lung during the surgery.^[5]^ The DLT ensures adequate ventilation, facilitates surgical procedure, and avoids high-pressure on suture lines.^[6]^

However, in our case, we used a single lumen tube (SLT) for lung isolation. The patient's diseased lung (Left side) was totally collapsed and not functioning; however, the other healthier lung (Right side) was functioning adequately enough to ventilate alone. Furthermore, there was no need to expand the surgical view by intentionally collapsing a lung. We report an unusual case of airway obstruction caused by thrombosis during a thoracic surgery using a SLT.

## 2 Case report

**Figure 1:**
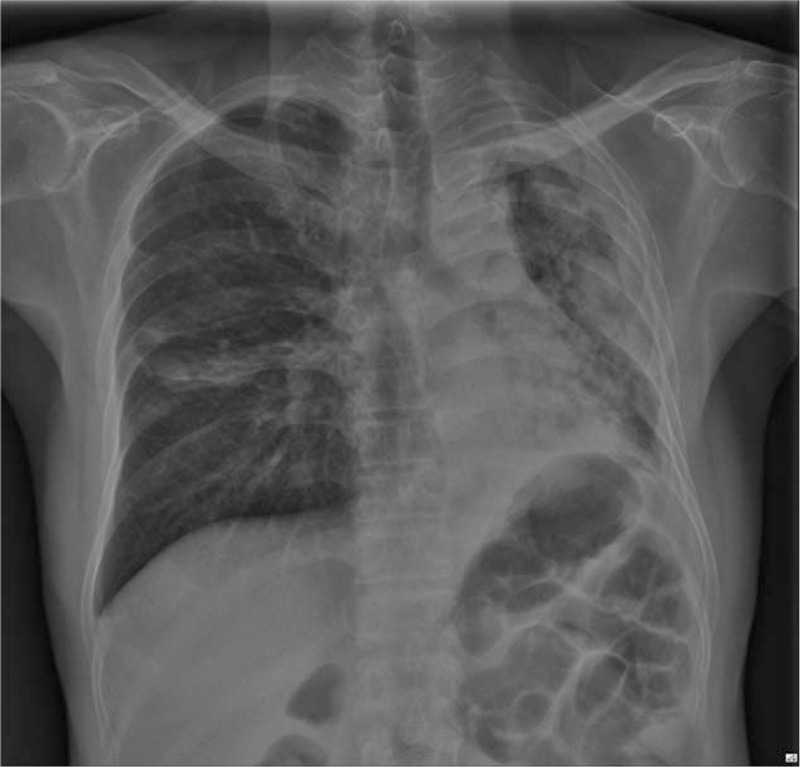
Preoperative chest x-ray (posteroanterior view).

**Figure 2:**
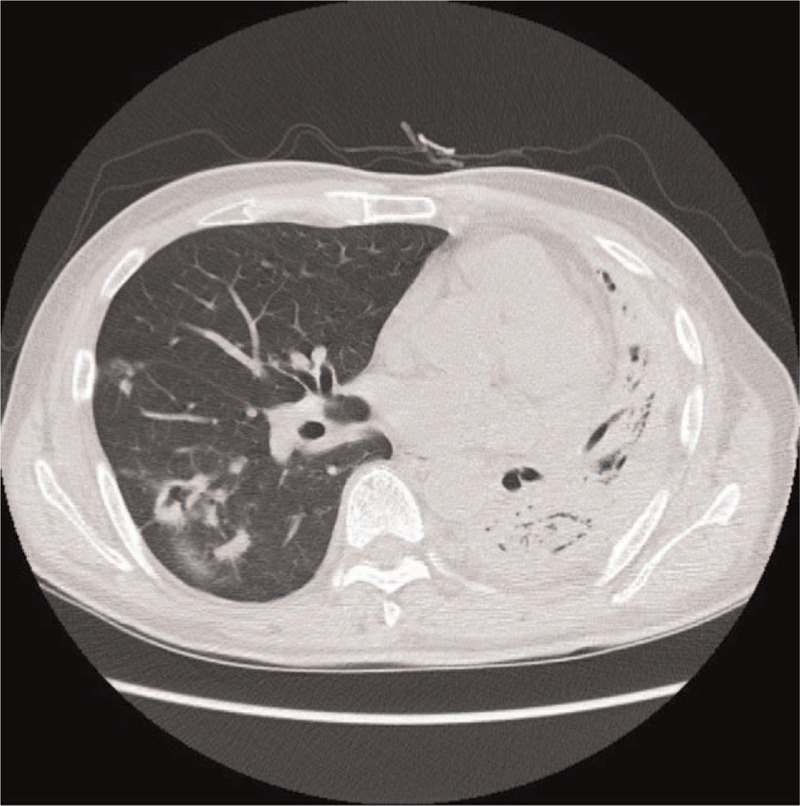
Preoperative chest computed tomography (axial view).

A 56-year-old man (164 cm, 57 kg) who was previously diagnosed with left Aspergilloma, a tuberculosis destroyed lung, and diabetes mellitus was scheduled for a left pneumonectomy due to recurrence of Aspergilloma in his left lung. As shown in his chest x-ray and chest CT (Figs. 1 and 2), his left lung was nearly collapsed, and the result of a pulmonary function test was severely restricted. The patient's diffusing capacity for carbon monoxide was 63% and predicted postoperative forced expiratory volume in 1 second was 23.5%, suggesting he might require a postoperative mechanical ventilator. However, the patient's preoperative room air saturation was 98% and arterial blood gas analysis (ABGA) showed pH 7.428, pCO_2_ 36.6 mmHg, pO_2_ 98.6 mmHg, and HCO_3_^-^ 24.9 mmol/L. As the patient's partial oxygen concentration was adequate despite his left lung being nearly totally collapsed, we thought that we would be capable of performing the pneumonectomy using a SLT. The thoracic surgeon also agreed to use a SLT. For a better surgical view, we planned lung isolation via insertion of a SLT deep into the bronchus.

**Figure 3:**
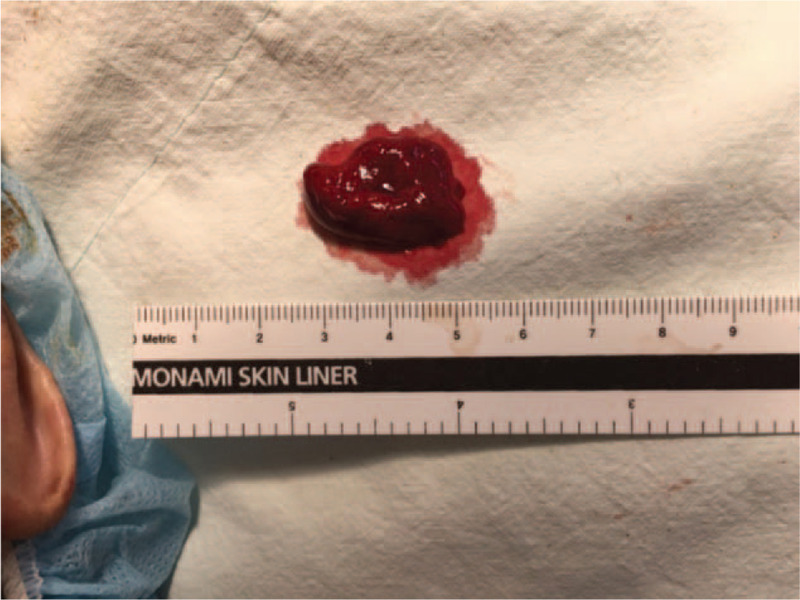
Hematoma from the single lumen tube (size: 2 cm).

On the day of the surgery, routine monitoring was done, including an electrocardiography, a noninvasive blood pressure measuring device, and pulse oximetry. Central venous line and arterial line were placed for better anesthetic care. Midazolam 2 mg, 2% propofol, and remifentanil were injected intravenously. After confirmation of unconsciousness, rocuronium 50 mg was injected for paralysis and a SLT was inserted into the patient's right bronchus. It was intubated deeper than the normal depth because we had to insert the tube into the right bronchus. After we checked via a fiberoptic bronchoscope and auscultated to confirm that the tube had been appropriately inserted, a silastic 7.0 sized single lumen tube (Covidien) was fixed 26 cm at the teeth. Total intravenous anesthesia was adjusted according to the bispectral index (BIS). The patient's position was changed from supine to right lateral decubitus and we reconfirmed through a fiberoptic bronchoscope that the tube was placed correctly. After opening the patient's thorax, the surgeon warned us that the patient's left lung had more adhesion than expected and therefore, a lot of bleeding could be expected. During the operation, there was excessive bleeding from the left lung, because of which we had to transfuse 2750 mL of packed red blood cells, 1200 mL of fresh frozen plasma, and 360 mL of platelets. We also carried out frequent tracheal suctions because there were large amounts of bloody secretions inside the tube. The patient's ABGA showed pH 7.425, pCO_2_ 37.7 mmHg, pO_2_ 169 mmHg, HCO_3_ 24.7 mmol/L with fraction of inspired oxygen 50% and his blood pressure was 109/57 mmHg, the heart rate was 72 beats per minute, and his oxygen saturation was 100%. After tracheal suction was performed, we tried a lung recruitment maneuver and suddenly end-tidal carbon dioxide did not show on the monitor. We tried manual-bagging without success. The patient's oxygen saturation was gradually dropping from 100% to 37%. Tracheal suction was performed and there was a bloody discharge but saturation did not improve. When we decided to use a bronchoscope to see what happened inside the tube, the patient's blood pressure dropped and heart rate was decreasing. We thought that cardiopulmonary resuscitation (CPR) was needed; therefore, 1 mg of epinephrine was injected via the central line. Surgeons stopped the procedure and started cardiac massage. The patient was in a right lateral decubitus position; hence, we moved him into a supine position and maintained suction through the trachea. During CPR, an approximately 2-cm-sized hematoma (Fig. 3) was removed from the endotracheal tube after vigorous suctioning. After removal of the hematoma, the ventilation and patient's vital signs recovered. To reduce risk, we reintubated the patient with a right-sided DLT and used a fiberoptic bronchoscope after intubation. After this event, the surgery was uneventful without any other complications. After surgery completion, the patient was transferred to an intensive care unit. Ten days after surgery, he was transferred to a ward and discharged on postoperative day 65 without any complications. The patient gave his permission to be included in the manuscript.

## 3 Discussion

According to previous studies, DLTs are the standard method of providing lung isolation in adults undergoing a pneumonectomy.^[7]^ However, we chose to use a single lumen tube because the patient's damaged left lung was totally collapsed and preoperative arterial blood gas was normal. In addition, the thoracic surgeon agreed with us that, because of the patient's repeated Aspergilloma and tuberculosis destroyed lung, the patient's diseased lung was so collapsed that there was no need to clear the view by using a DLT. After the surgery, he needed a delayed extubation and a mechanical ventilator using a single lumen tube so it would be better to use a single lumen for the first choice. We anticipated that if we inserted a SLT deep into the patient's right bronchus, the right lung would not be affected by the collapsed lung. Furthermore, we examined him by auscultation and confirmed the breathing sound on the right side of the lung. We also checked through a fiberoptic bronchoscope after the position change to confirm that the tube was placed correctly. However, we missed the perioperative risk of a pneumonectomy such as intraoperative spillage. According to a study, the overall operative mortality for the first 30 days after pneumonectomy ranges from 5% to 13%.^[5]^ Since there was excessive bleeding of the patient's lung due to adhesions, we did not think that a blood clot could have formed on the upper side of the patient's left lung. Bloody secretion was present in the tube; hence, we considered the possibility that the bloody secretion was infusing into the trachea. Since we maintained suction in the tube, the tube could have moved gradually up into the trachea and the incident occurred when the hematoma totally blocked the tube. This explains the airway obstruction caused by a blood clot moving from the surgical field into the trachea. Since the blood clot blocked the single tube, there was no way to ventilate with oxygen which is why the patient's saturation was decreasing. Manual-bagging was also impossible with the total occlusion. Had we used a DLT, the blood clot that was formed in the patient's collapsed lung would not have been able to move through the endotracheal tube and would not have blocked the airway. An alternative technique would be to use a bronchial blocker. The bronchial blocking technique involves blockade of the mainstem bronchus to allow lung collapse distal to the occlusion.^[8]^ However, there is a possibility of obstruction due to migration of the blocker or the endotracheal tube itself. Besides, pneumonectomy requires the closure of left main bronchus stump and to do that, we have to get rid of the tube meaning there is a little chance of preventing a hematoma coming from a surgical field. We assumed that because of the adequate function of the uncollapsed lung, it would be reasonable to use a single endotracheal tube. However, we cannot exclude the possibility of severe adhesions, bleeding, or soiling from the abscess. Moreover, there are not many cases using a single tube during pneumonectomy; therefore, we found this case meaningful to warn others.

## 4 Conclusion

In conclusion, intraoperative airway obstruction can result from rare causes, such as thrombosis. We conclude that it is important to use a DLT for the patient's safety during thoracic surgery.

## Author Contributions

**Conceptualization:** Shill Lee Son, Miyoung Kwon, Yeon-Jin Moon.

**Data curation:** Shill Lee Son, Woo-Shik Kim, Yeon-Jin Moon.

**Formal analysis:** Miyoung Kwon, Yeon-Jin Moon

**Resources:** Shill Lee Son, Yeon-Jin Moon

**Supervision:** Miyoung Kwon, Yeon-Jin Moon

**Validation:** Miyoung Kwon, Yeon-Jin Moon

Shill Lee Son orcid: 0000-0001-9738-3973.
